# Power in numbers: Harnessing global data to unravel the alcohol-pancreatic cancer link

**DOI:** 10.1371/journal.pmed.1004615

**Published:** 2025-05-22

**Authors:** Gilaad G. Kaplan

**Affiliations:** Departments of Medicine and Community Health Sciences, University of Calgary, Calgary, Alberta, Canada

## Abstract

In this Perspective, Gilaad G. Kaplan discusses the power of harmonizing global data to investigate associations between alcohol consumption and pancreatic cancer risk.

## Teaser

The global burden of pancreatic cancer is substantial, highlighting the need to identify modifiable risk factors. In a multi-country study, Sabine Naudin and colleagues demonstrate a clear association between alcohol consumption and the risk of pancreatic cancer.

The burden of pancreatic cancer is considerable, accounting for 3% of all cancer cases and 6% of cancer-related deaths in the European Union [1]. Pancreatic cancer is a leading cause of cancer-related mortality due to the difficulty in detecting early-stage disease and the limited treatment options available for those diagnosed at advanced stages [1]. With no widely recommended screening programs for pancreatic cancer, preventive health strategies are essential to reducing the burden of pancreatic cancer. Specifically, identifying modifiable environmental risk factors that can inform both personal decisions and population-level policies is crucial [2].

One such factor, extensively studied with variable results, is the impact of alcohol consumption on pancreatic cancer incidence [2]. Differences in prior findings have largely been attributed to heterogeneity in study populations, designs, and analytical approaches [3,4]. Sabine Naudin and colleagues have compiled prospective cohorts from around the world to provide the most comprehensive study to date on the association between alcohol exposure and pancreatic cancer risk [5]. The significance of this research lies in equipping the global community with the evidence necessary to mitigate, in part, the burden of pancreatic cancer.

The authors analyzed data from 30 population-based prospective cohorts across four continents: North America, Europe, East Asia, and Australia [5]. The study pooled individual-level data from 2,494,432 participants without a prior cancer diagnosis, recruited between 1980 and 2013, with a median follow-up of 16 years, during which 10,067 new cases of pancreatic cancer were identified. Compared to individuals consuming 0.1– < 0.5 g/day of alcohol, those consuming 30–60 g/day had a 12% higher risk of developing pancreatic cancer, while those consuming more than 60 g/day had a 32% higher risk. Each additional 10 g/day of alcohol intake was associated with a 3% increase in pancreatic cancer risk. Although sex did not modify this relationship, threshold levels were identified, with the highest risk observed among women consuming 15 g/day or more and men consuming at least 30 g/day. Notably, the type of alcohol appeared to influence pancreatic cancer risk, with significant associations observed for beer and liquor consumption, but not for wine. Geographic differences were also evident, as the association between alcohol intake and pancreatic cancer risk was not observed in East Asian cohorts [5]. Collectively, these findings highlight the role of alcohol consumption as a risk factor for pancreatic cancer while underscoring the importance of nuanced considerations in assessing individual risk and shaping public health policies.

A key strength of this study was the use of an international consortium that pooled individual-level data from 30 distinct cohorts. However, these prospective cohorts were originally designed as dietary studies and were secondarily used to evaluate the relationship between alcohol consumption and pancreatic cancer risk, introducing potential heterogeneity due to methodological differences [6]. To mitigate this variability, the investigators established strict inclusion criteria, selecting only cohorts that collected daily alcohol intake in grams of ethanol per day and met minimum thresholds for both alcohol exposure (i.e., at least 10% of the sex-specific sub-cohort reporting alcohol intake ≥0.1 g/day) and cancer outcomes (i.e., a minimum of 50 incident pancreatic cancer cases during follow-up) [5]. The approach of harmonizing across studies and pooling individual-level data across cohorts represents a significant advancement over traditional meta-analyses that aggregate study-level data [6]. However, as with all observational studies, inherent biases—such as residual confounding, misclassification error, and selection bias—persist. As a result, while the study provides valuable insights, cautious interpretation of its findings remains necessary [7].

Studying the complexities of alcohol consumption—including dose, duration, and patterns of exposure—is important for clarifying the underlying biological mechanisms of pancreatic cancer development from potential methodological biases. One example of the study’s methodological challenges was the decision to use 0.1–5 g/day of alcohol consumption as the referent category, rather than individuals who rarely or never drank alcohol. Notably, men who self-reported consuming <0.1 g/day had a higher risk of pancreatic cancer than those consuming 0.1–5 g/day [5]. This choice underscores a common limitation in observational environmental health research, where self-reported questionnaires may misclassify exposure or fail to account for prior heavy drinkers who may have quit before enrollment in a cohort [7]. While key confounders such as smoking were adjusted for, other factors such as chronic pancreatitis—associated with both alcohol consumption and pancreatic cancer risk—were not available within the cohorts [8,9].

Additionally, the study’s alcohol-specific findings, which demonstrated an increased risk associated with beer consumption but no association with wine, may reflect underlying differences in the type of alcohol itself. However, these variations may also indirectly highlight the complex interactions of social, cultural, and economic factors that observational studies struggle to disentangle [10]. Heavy beer consumption, for example, may be more prevalent among individuals of lower socioeconomic status, who are more likely to also engage in higher-risk lifestyles, including poor dietary habits [11]. Conversely, wine consumption is often associated with wealthier individuals who may have greater access to healthier diets and lifestyles [11].

Given these complexities, examining alcohol consumption in isolation may be less informative than assessing composite measures of lifestyle factors. Approaches such as the healthy lifestyle scores, which integrates alcohol consumption with physical activity, body mass index, smoking, and adherence to a Mediterranean-like diet, may provide a more comprehensive understanding of risk factors [2]. Moreover, recognizing the importance of genetic predisposition, future prospective cohort studies that integrate genetic risk scores with composite measures of modifiable environmental risk factors, including alcohol consumption, offer an opportunity to identify high-risk groups who may benefit from targeted pancreatic imaging for early detection [2].

This study represents the most comprehensive multi-country prospective cohort analysis to date, providing robust evidence of an association between heavy alcohol consumption and an increased risk of developing pancreatic cancer—a disease with significant morbidity and mortality. Adding to the extensive body of research linking high alcohol intake to various health risks, from carcinogenesis to liver cirrhosis, this study serves as a call to action for patients, healthcare providers, public health officials, and policymakers. The identification of threshold levels in this study suggests that simple interventions—such as eliminating the largest serving size of beer and wine, which has been shown to lower overall alcohol use—may lead to significant public health benefits [12,13]. Establishing clear guidelines and recommendations to limit alcohol consumption has the potential to reduce the global burden of pancreatic cancer and improve overall health outcomes.

Meanwhile, future research can further explore nuanced differences based on the type of alcohol, specific subpopulations, and generalizability across geographic regions. Finally, this study serves as a model for environmental health research, demonstrating the value of international consortia that harmonize study design and analysis. By pooling individual-level data across diverse global cohorts, researchers can better elucidate the fundamental relationships between environmental exposures and health outcomes across varied populations.
